# Impact of animal source on biocompatibility of bone xenografts: A comparative animal study

**DOI:** 10.34172/japid.025.3443

**Published:** 2025-06-11

**Authors:** Ardeshir Lafzi, Reza Amid, Mahdi Kadkhodazadeh, Anahita Moscowchi, Javad Mehrani, Nima Ahmadi, Amirali Karimi Vasigh

**Affiliations:** ^1^Dental Research Center, Research Institute for Dental Sciences, Shahid Beheshti University of Medical Sciences, Tehran, Iran; ^2^Department of Periodontics, School of Dentistry, Shahid Beheshti University of Medical Sciences, Tehran, Iran; ^3^Department of Periodontics, Faculty of Dentistry and Tissue Engineering, School of Advanced Technologies in Medicine, Mazandaran University of Medical Sciences, Sari, Iran

**Keywords:** Antler, Bone substitute, Cytotoxicity test, Irritation testing, Sensitization testing

## Abstract

**Background.:**

The study aimed to compare the results of cytotoxicity and in vivo irritation and sensitization tests of a new antler-derived bone substitute with those of a bovine-derived xenograft.

**Methods.:**

This study included an in vitro cytotoxicity test based on ISO 10993‐5 standard. Additionally, in vivo irritation and sensitization tests were carried out according to ISO 10993-10 standard protocol.

**Results.:**

The cytotoxicity test showed a viability of 99.46±1.09% for the antler-derived bone substitute and 98.42±1.84% for the bovine xenograft (*P*=0.445). Furthermore, after 24 hours, no differences in morphological grade were found in both samples. The irritation test indicated a primary irritation index (PII) score of 0 for both the antler and bovine xenografts. Likewise, the sensitization test demonstrated a sensitization score of 0 for both the antler and bovine xenografts. All animals appeared clinically normal throughout the study in both in vivo tests, and all sites of the test extract and the reagent control seemed normal.

**Conclusion.:**

Both the antler-derived and bovine xenografts were found to be non-toxic, non-irritating, and non-sensitizing. Further studies should be conducted on other essential laboratory tests and animal and clinical studies.

## Introduction

 Bone augmentation is a method used to reconstruct the alveolar ridge. The procedure aims to create a well-vascularized bone structure capable of natural remodeling. In clinical settings, four main types of bone grafting materials are available: autogenous bone, allografts, xenografts, and alloplasts.^1,2^ Although autogenous bone is the gold standard, its use is limited by low intraoral bone quantities and patient morbidity.^3^ As a result, using non-autogenous bone grafts has gained popularity. However, allografts have limitations, including the potential for disease transmission, immunological reactions, and the absence of the bone-inducing properties of autografts.^4,5^

 Xenografts are a popular option due to their ready availability from various sources. Additionally, they offer the advantages of more accurate sterilization and lower costs. Nevertheless, it is noteworthy that bovine xenografts may transmit common diseases of cattle and humans.^6,7^ Thus, introducing safer, effective, and ethically acceptable xenografts is desirable.

 Deer antlers are a viable alternative to human bone since they are the only mammalian organs that can regenerate completely independently because of their favorable blood flow and structural features that are similar to human bone.^8,9^ Moreover, since the deer antler is a non-vital organ, creating a xenograft from it will be easier.

 Various tests, such as cytotoxicity tests, physical and chemical structure studies, and animal experiments, must be conducted to confirm the effectiveness of xenografts. Since xenografts come from different species, it is crucial to check material safety for cytotoxicity, irritation, and sensitization potential.^9,10^ Cytotoxicity testing involves assessing the ability of certain chemicals or mediator cells to destroy living cells. Using a cytotoxic compound, healthy living cells can be induced to undergo necrosis or apoptosis.^11,12^ Additionally, irritation testing is essential in evaluating the potential to cause an immediate irritation reaction following exposure to the body. The sensitization test determines the sensitizing activity of chemicals and medical devices. By conducting this test, the potential of a material or product to cause a delayed hypersensitivity reaction can be determined.^13^

 Even though deer antlers have the potential to be efficient xenografts, they are not yet produced commercially in Iran as a routine bone graft substitute. Therefore, the Technology Unit of the Research Institute for Dental Sciences at Shahid Beheshti University of Medical Sciences has created a xenograft derived from Persian gazelle antlers to make a safe and efficient xenograft. Considering the lack of data about antler laboratory tests, this study aims to evaluate the safety of a novel Persian gazelle antler-derived xenogeneic graft in terms of cytotoxicity, irritation, and skin sensitization.

## Methods

 This study was carried out at Nikoopharmed laboratory in Tehran, Iran, with approval from the Ethics Committee of Shahid Beheshti University of Medical Sciences (IR.SBMU.DRC.REC.1400.148).

###  Sample preparation

 The test and control samples used in this study were extracted from deer antler (Maral Pajoohesh Shams, Iran) and bovine bone (Bone ^+^ B^®^, Novateb, Iran), respectively, with the latter taken from the femur region. Both samples were prepared using chemical and thermal techniques and sterilized with gamma irradiation for testing cytotoxicity and irritation under ISO 10993-10 (https://www.iso.org/standard/40884.html) and ISO 10993-12 (https://www.iso.org/standard/75769.html) standard protocols. The extraction was done under 37 ± 1 °C for 72 ± 2 hours with an extraction ratio of 0.2 g/mL ± 10% in a dynamic environment. The sample extraction took place in an environment with a temperature of 20 ± 2 ºC.

###  Cytotoxicity test

 The cytotoxicity test was conducted using the 3-(4,5-dimethylthiazol-2-yl)-2,5-diphenyltetrazolium bromide assay (MTT assay) under ISO 10993-5 standard method (https://www.iso.org/standard/36406.html), for both the test and control samples. The MTT compound (3-(4, 5-dimethylthiazol-2-yl)-2,5-diphenyl-2H-tetrazolium bromide) was used to execute the cytotoxicity test. L929 mouse fibroblast NCTC clone 929 strain L was selected as the cell line and maintained in the minimum essential medium (MEM) at a constant temperature of 23 ± 3 ºC. The testing protocol involved seeding 96-well plates initially and incubating them in 37 °C/5% CO_2_ for 24 ± 2 hours, followed by treatment of the plates with test sample extract of ≥ 4 concentrations in the treatment medium (100 μL). Morphological alterations of the cells were then examined using microscopic evaluation. Later, 50 μL of MTT solution was added to each well, and the plates were incubated at 37 °C/5% CO_2_ for 2 hours. After removing the MTT solution, 100 mL of isopropanol was added to each well of the plate, and the absorption rate was analyzed at 570 nm (reference 650 nm).

###  Irritation test

 To conduct the in vivo irritation test for both test (antler xenograft) and control (bovine xenograft) samples, the ISO 10993-10 standard method was followed. Three albino rabbits with intact, healthy skin weighing 2.3‒2.6 kg were selected for each sample to perform the test. Before the treatment, the healthy animals were acclimatized to the laboratory conditions. Then, they were housed individually in stainless steel suspended cages with a card attached indicating the identification number of the test material and the first treatment date. The temperature and humidity of the room were monitored daily. Only healthy animals not previously used in any tests were chosen for the experiment.

**Figure 1:**
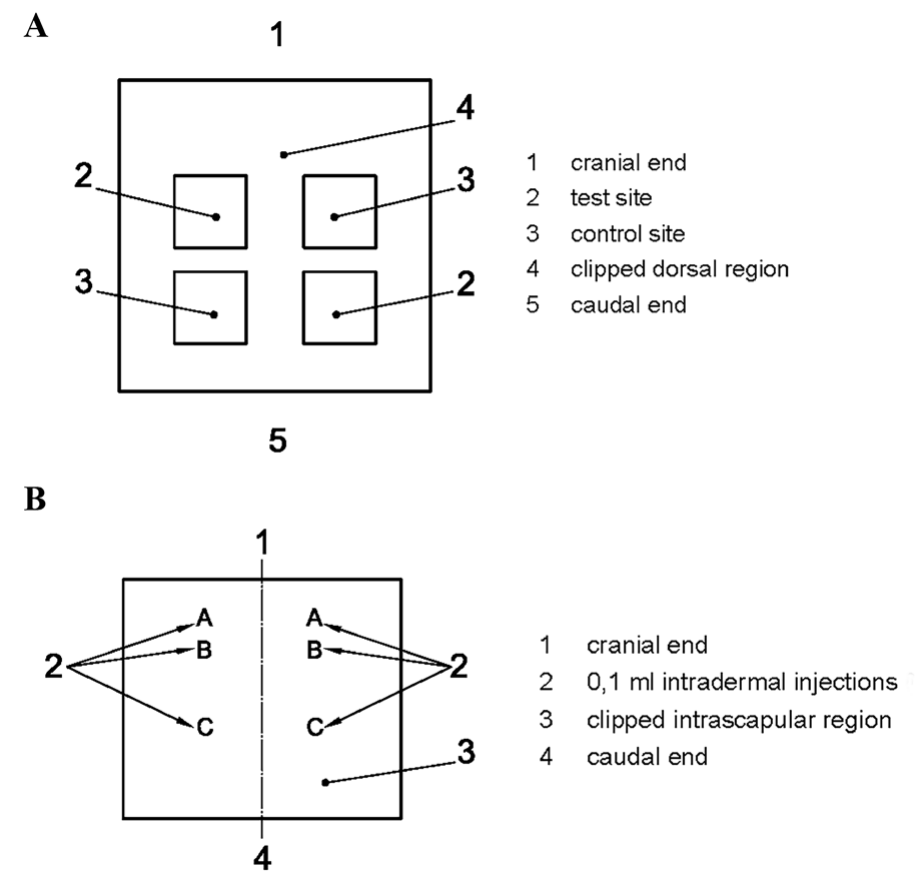
Location of skin application sites for (A) irritation test and (B) sensitization test

 The irritation test process involved the following key steps. Firstly, the back of the animal models was prepared to provide sufficient distance on both sides of the spine for application and observation of all test sites (approximately 10 × 15 cm). Secondly, a test sample and negative control were applied based on the experimental design after extracting the samples, as indicated in Figure 1. Then, the animal models were observed under natural, full-spectrum lighting to visualize skin reactions. The skin reactions in terms of erythema and edema were described and scored according to the system given in Table 1. Finally, the animal models were evaluated at 24 ± 2 hours, 48 ± 2 hours, and 72 ± 2 hours to calculate the primary irritation index (PII). The irritation index is given in terms of a number (score) and description (response category) as follows: negligible (0 to 0.4), slight (0.5 to 1.9), moderate (2 to 4.9), and severe (5 to 8).

###  Skin sensitization test

 The in vivo sensitization test for both test and control samples was conducted based on the ISO 10993-10 standard method. For each sample, 15 albino Guinea pigs (10 treated animals and 5 control animals) with a body weight of 300‒500 g were used for the test. The animals’ housing, environment, and selection criteria were similar to those in the irritation test. The back of the animal models was prepared to provide sufficient distance on both sides of the spine for application and observation of all test sites (approximately 10 × 15 cm). The samples were then extracted, and a test sample and negative control were applied based on the experimental design, as shown in Figure 1.

 The test was carried out through three phases: intradermal induction phase, topical induction phase, and challenge phase. Each animal received two 0.1-mL intradermal injections during the intradermal induction phase. These injections were administered at the designated injection sites on the intrascapular region, as shown in Figure 1. Site A was injected with a 50:50 volume ratio stable emulsion of Freund’s complete adjuvant mixed with the selected solvent, site B was injected with the undiluted test sample, and the control group was injected with the solvent alone. At site C, the test sample was injected at the concentration used for site B, emulsified in a 50:50 volume ratio stable emulsion of Freund’s complete adjuvant and the solvent (50%). The control animals were injected with an emulsion of the blank liquid with the adjuvant.

 During the topical induction phase, which took place 7 ± 1 days after the intradermal induction phase, a patch of approximately 8 cm^2^ (in size) containing the test sample was topically applied to the intrascapular region of each animal, making sure to cover the intradermal injection sites, using either filter paper or absorbent gauze.

 In the challenge phase, which was conducted 14 ± 1 days after the topical induction phase, all control and test group animals were challenged with the test sample. The test sample and a blank were applied through topical application on sites that were not treated during the induction phase, for instance, the upper flank of each animal, using appropriate patches or chambers soaked in the test sample at the concentration selected in the “intradermal induction phase” for site C.

 The test and control animal sites were examined 24 ± 2 hours and 48 ± 2 hours after removing the dressings. The skin reactions, including erythema and edema, were assessed using Magnusson and Kligman grading standards. These standards involved assigning a score of 0 for no visible change, a score of 1 for discrete or patchy erythema, a score of 2 for moderate and confluent erythema, and a score of 3 for intense erythema and/or swelling. The test group’s Magnusson and Kligman scores were compared to those of the control animals to determine sensitization. Sensitization was present if the test group received a score of ≥ 1 and the control group scores were < 1. However, if any score in the control group was ≥ 1, then the test animal’s reactions exceeding the most severe response in the control animals were presumed to result from sensitization.

###  Statistical analysis

 MTT data are presented as mean ± standard deviation (SD) values. The normality of the distributions was assessed using the Shapiro-Wilk test. As the data were normally distributed, they were compared using independent t-test (*P* < 0.05 was considered significant at a 95% confidence interval). Statistical analyses were conducted using SPSS 26 (SPSS Inc., IL, USA).

## Results

###  Cytotoxicity test

 The L929 mouse fibroblast NCTC exhibited a 99.46 ± 1.09% (95% CI: 96.74‒102.2) viability rate for the antler xenograft’s in vitro cytotoxicity response, while the control group product showed a 98.42 ± 1.84% (95% CI: 93.84‒103) viability rate after 24 hours, indicating no statistically significant difference between the groups (*P* = 0.445).

**Figure 2:**
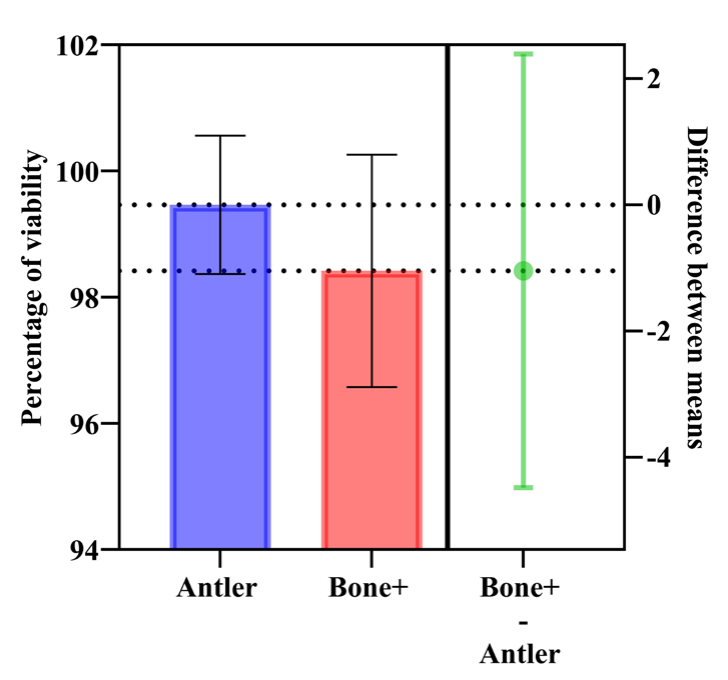
Results of cytotoxicity test: MTT assay after 24 hours in antler-derived and bovine xenografts

 Furthermore, no changes in morphological grade were observed in either sample during the 24-hour assessment period (Figure 2).

###  Irritation test 

 The animals treated with both antler-derived and bovine xenografts exhibited no clinical abnormalities throughout the study. The test extract and reagent control sites showed no signs of damage. As seen in Table 2, the PII of the test article for both groups was 0.0. Additionally, the irritation response category in rabbits for both antler-derived and bovine xenografts was 0, suggesting a negligible mean score for the 72 ± 2 hours assessment period.

**Table 1:** Scoring system for skin reaction in irritation test

**Reaction**	**Irritation score**
Erythema and scar formation	
No erythema	0
Very slight erythema (barely perceptible)	1
Well-defined erythema	2
Moderate erythema	3
Severe erythema (beet-redness) to eschar formation preventing grading of erythema	4
Edema formation	
No edema	0
Very slight edema (barely perceptible)	1
Well-defined edema (edges of area well-defined by definite raising)	2
Moderate edema (raised approximately 1 mm)	3
Severe edema (raised more than 1 mm and extending beyond exposure area)	4
Maximal possible score for irritation	8

**Table 2:** Results of the irritation test

**Animal No.**	**Group**	**Site**	**Acceptable limit**	**Irritation score**						**Results**
				**Erythema**			**Edema**			**Average score**	**Pass/Fail**
				**24±2**	**48±2**	**72±2**	**24±2**	**48±2**	**72±2**		
1	Test sample	A	0 – 0.4	0.0	0.0	0.0	0.0	0.0	0.0	0.0	Pass
		B		0.0	0.0	0.0	0.0	0.0	0.0		
	Control	A		0.0	0.0	0.0	0.0	0.0	0.0	0.0	Pass
		B		0.0	0.0	0.0	0.0	0.0	0.0		
2	Test sample	A	0 – 0.4	0.0	0.0	0.0	0.0	0.0	0.0	0.0	Pass
		B		0.0	0.0	0.0	0.0	0.0	0.0		
	Control	A		0.0	0.0	0.0	0.0	0.0	0.0	0.0	Pass
		B		0.0	0.0	0.0	0.0	0.0	0.0		
3	Test sample	A	0 – 0.4	0.0	0.0	0.0	0.0	0.0	0.0	0.0	Pass
		B		0.0	0.0	0.0	0.0	0.0	0.0		
	Control	A		0.0	0.0	0.0	0.0	0.0	0.0	0.0	Pass
		B		0.0	0.0	0.0	0.0	0.0	0.0		
Total average of test samples				0.0	0.0	0.0	0.0	0.0	0.0	0.0	Pass

###  Skin sensitization test

 During the study, no animals treated with antler-derived or bovine xenografts exhibited any clinical abnormalities. Furthermore, no damage was observed at the test extract or reagent control sites. Both the test extract and reagent control scores were 0 for both groups. Additionally, the sensitization score of the test material was also 0 for both antler-derived and bovine xenografts.

## Discussion

 Xenografts intended for use as bone substitutes must meet specific criteria, including biocompatibility, non-toxicity, and non-immunogenicity. These qualities are determined through various tests, such as cytotoxicity, irritation, and sensitization assays. Our research findings have indicated that both antler-derived and bovine xenografts are non-toxic, non-irritating, and non-sensitizing. Consequently, these xenografts offer a safe and effective bone substitute solution.

 Several techniques are available to replace localized bone loss, including the use of autogenous and allogeneic bone. While these methods have been successfully implemented in the past, obtaining autogenous bone grafts carries risks, and the amount of available bone is limited.^14^ Likewise, allografts have a history of significant postoperative infection and fracture rates, and there is a potential risk of disease transmission.^15,16^ Therefore, alloplastic materials and xenografts have been developed as additional options for bone replacement and defect filling.^17^ Xenografts are especially appealing to clinicians due to their inorganic structure, which is similar to deproteinized human bone in their porous architecture and composition. They are extensively available and have demonstrated satisfactory efficacy in repairing and healing bone defects.^7,18^ Xenografts can be obtained from various species, including cows, pigs, camels, and ostrich. However, the majority of available xenografts require animal sacrifice, resulting in animal ethics and welfare concerns. Therefore, it is critical to develop a xenograft that can be produced from an animal’s regeneratable parts. Antlers are an excellent example of a suitable model for a xenograft due to their regenerative ability and material characteristics.^9^ Antlers do not contain living cells or fat, simplifying the preparation process for antler xenografts.

 Cytotoxicity testing is a vital pilot project test and a crucial indicator for evaluating the toxicity of medical devices. It is a fast and sensitive method that can help save animals from toxicity issues.^19^ Testing biomaterials for their cytotoxicity is vital in assessing their safety on both target and off-target cells. Conducting toxicity testing for on- and off-target effects is crucial for safely administering bone substitutes. In vitro assessments help establish the toxicity of a biomaterial compound in the human body.^20^ In this survey, the results of cell death percentage showed that the percentage of cell death in both xenografts was < 30% after 24 hours, suggesting that the investigated antler-derived xenograft did not cause cytotoxic effects on fibroblasts and instead encouraged the growth of more fibroblasts.

 The irritation test is useful for predicting the acute skin irritation potential of chemicals or substances. This assay can be used to evaluate the dermal irritation potential of medical devices that may contain low concentrations of irritants and differentiate between irritants and non-irritants. Moreover, it is an ethical, valid, and reliable assay that has been validated for its biological relevance.^21^

 Skin sensitization testing is crucial for identifying substances that may cause allergic contact dermatitis.^22^ Currently, three animal assays are available to assess the skin-sensitizing potential of chemicals: two guinea pig assays and one murine assay. The guinea pig maximization test (GPMT) and the Buehler test or closed-patch test are the two most commonly used methods for testing skin sensitivity. Among these, the maximization test is the most sensitive.^23^ For our study, we used the GPMT as it is well-suited for testing substances that may be in contact with the skin. The closed-patch test is more appropriate for topical products.

 It is important to highlight that another investigation into the residual solvents and sterility of the prepared grafting material has confirmed its safety.^24^ In addition, the potential for tissue regeneration can be significantly increased by using non-toxic biomaterials that do not trigger immediate or delayed immunological responses. Additionally, precise assessment of cytotoxicity, irritation, and sensitization reactions can be critical in identifying compounds that could pose health risks to humans. This is particularly crucial during the research stage of creating new bone substitute products to ensure user safety.

## Limitations

 We only carried out three tests out of the full set of tests required to verify the clinical use of xenografts. Furthermore, we did not examine how the preparation methods impact clinical outcomes or the induction of osteogenic factors; for instance, we did not compare the cell response to bone graft substitutes prepared through various methods.

## Conclusion

 The present study demonstrated that the xenograft derived from antlers was properly prepared without toxicity, irritation, or sensitization. However, more comprehensive investigations involving animal and clinical studies and additional in vitro research involving various laboratory tests are warranted.

## Competing Interests

 The authors declare that they have no competing interests.

## Consent for Publication

 Not applicable.

## Data Availability Statement

 The data will be shared at a reasonable request by the corresponding author.

## Ethical Approval

 This study was approved by the Ethics Committee of Shahid Beheshti University of Medical Sciences, Tehran, Iran (IR.SBMU.DRC.REC.1400.148).
